# Chinese universities’ special programs supporting talents to seek a United Nations career: a center-periphery-model analysis

**DOI:** 10.1007/s10734-022-00938-1

**Published:** 2022-10-14

**Authors:** Suyu Liu, Wenjun Ding

**Affiliations:** 1 Zhongnan University of Economics and Law, Wuhan, China; 2 School of Education, University of Bristol, Bristol, UK

**Keywords:** Center-periphery model, United Nations, Chinese higher education, Special programs

## Abstract

In recent years, China’s higher education sector has started to establish special programs to train and support talents to seek career opportunities in the United Nations (UN). To explore these special programs and understand their relationship with China’s internationalization strategies and its higher education, we used the center-periphery model as the theoretical framework. We analyzed 53 institutional documents and conducted semi-structured interviews among 5 university staff members and 21 students/recent graduates who were involved in these special programs. The analysis on the special programs implied Chinese higher education’s peripheral position in supporting talents to work in the UN. This was reflected by the conforming practice, including accepting current UN recruitment regulations and English’s dominance in the UN recruitment practice. However, we also identified alternative dynamics that China and its higher education do not simply obey the center-periphery model and accept their peripheral status. Instead, special programs were established to achieve China’s global strategy of moving to the center of international arena via multilateralism and international organizations such as the UN. This study sheds light on further explorations of the state-university relationship in China in the globalization era, especially from the perspective of cultural diplomacy and soft power.

## Introduction

The center-periphery (core-periphery) model is a widely used analytical framework for studies on globalization and internationalization. The interpretation of the world as a center-periphery structure can be traced back to Raul Prebisch’s dependency theory about world economy (Marginson & Xu, 2021; Rojas, 2013; Wallersterin, 2004). This model has been further developed in the field of economic geography and international trade and provides explanations to resource re-distribution and concentration, inequalities of development, and cross-national competition in the era of globalization (Krugman, 1991). As argued by Krugman and Venables (1995), in a globalized world, the center is mainly taken up by industrialized economies while less industrialized economies are largely on the periphery. Countries in the center enjoy the advantage of industrial agglomeration in the globalization, such as more skilled labor (Forslid & Ottaviano, 2003). Countries in the periphery, with the declining transport cost, may also benefit from the re-industrialization and income-generation opportunities brought by globalization. China’s position in the center-periphery model has attracted scholars’ attention. For example, China’s starting position in the globalization was the periphery, but with its rapid economic development, it is moving towards the center (Cheng & Zhai, 2021).

The application of the center-periphery model has been extended to the field of higher education internationalization (e.g., Altbach, 2009). As argued by Altbach (2009) and Xu (2020), higher education institutions (HEIs) in the industrialized countries, mostly English-speaking countries, are usually in the core position. They are the main producers and disseminators of knowledge, and the main developers of norms and hierarchy in higher education, which are obeyed by HEIs in the peripheral countries (Wallerstein, 2000; Xu, 2020). Similar to the situation in economic geography, in the higher education internationalization, countries on the periphery may have incentives to move towards the center.

We can find that China is still on the periphery when applying the center-periphery model to interpret higher education’s special programs of supporting talents to seek job opportunities in the United Nations (UN). By comparison, countries with a higher level of economic and educational development such as the USA, France, Switzerland, and Germany are located in the center. China’s peripheral position in the center-periphery hierarchy can be reflected by the relatively small number of UN employees with Chinese nationality and the late establishment of special programs in China’s HEIs of training suitable talents to seek career opportunities in the UN.

In face of China’s perceived weak international influence due to the insufficient number of Chinese nationals working in the UN, the Chinese government has started to train and support more suitable talents to apply for career opportunities in international organizations (especially the UN) since 2010. This is part of its national development strategy and “going-out” internationalization strategy (Ministry of Education, 2010). The initial emphasis was on the direct selection and recommendation of suitable talents from the state sector, especially the government, to work in the UN. This practice gradually started to include supporting and training potential candidates to apply for UN career opportunities by enhancing their own competencies which are required by the UN (Chinese Communist Party Central Committee & China State Council, 2015). Therefore, HEIs, the major stakeholder that trains potential candidates, have started to play a more important role.

In recent years, the Chinese higher education sector, especially the HEIs, has established a variety of special programs to train and support suitable candidates to work, including taking internships, in the UN.^1^ Such a trend has been on the rise especially after Chinese President’s official visit to the headquarter of United Nations Educational Scientific and Cultural Organization (UNESCO) in 2014. These programs aim to encourage more young Chinese nationals, especially university students/graduates and junior scholars, to seek career opportunities in the UN.

Despite the burgeoning of these special programs, relevant studies on them are very limited. Some fundamental questions remain unanswered: What are these programs? Why do students/recent graduates participate in these programs? Why do universities establish these special programs? Moreover, there is a lack of research on the relationship between these special programs and the internationalization strategies of the country and the higher education sector.

To address these research gaps, this study uses the center-periphery model to explore the following research questions:

1. What are these special programs, and what is the experience of the participants of these special programs?
2. How are these special programs integrated into the country and higher education sector’s internationalization strategies?

Answers to these questions will fill in our knowledge gap of the Chinese special programs that support talents to work in the UN and contribute to the interpretation and application of center-periphery model in the internationalization of higher education. These questions also enable a further exploration of the state-university relationship in China in the globalization era, especially from the perspective of cultural diplomacy and soft power (Yuan, 2014). Before moving to the details on research methods, this paper further introduces the application of center-periphery model in higher education internationalization and supplying talents to the UN, as well as the internationalization of Chinese higher education sector.

## Literature review

### Center-periphery model and the special programs

The center-periphery model outlines and explains the advantages gained by countries located in the center, including the privileges in higher education (Xu, 2020). Regarding China, Altbach (2001) argued that it remained on the periphery, especially its higher education which is largely based on western modes. Nevertheless, China’s rapid progress in higher education and increasing efforts to expand international influence are noted. Using a more flexible approach, Cheng and Zhai (2021) suggested that China is in the quasi-periphery position and advancing towards the core. In higher education sector, this can be reflected by China’s graduate shift to a more “outward-oriented” higher education (Wu, 2019), rather than merely learning from the west (Xu, 2020).

In terms of the number of citizens working in the UN, China can be considered as on the periphery of the UN system. Liu and Xiao (2018) found that according to China’s membership fee contribution to the UN, the minimum quota for Chinese employees in the UN Secretariat in 2015 should be 119, but only 71 Chinese nationals were employed in the UN Secretariat in 2015. Recent data from the UN System Chief Executives Board for Coordination (2021) showed that, although the number of Chinese staff in the UN increased to 1384 in 2020, it only took up 1.2% of the total. In a sharp contrast, there were 5459 US nationals working for the UN in 2020, accounting for 4.7% of the total employed personnel. This is followed by France with 4364 UN employees in the same year, taking up 3.7 of the total (UN Chief Executives Board for Coordination, 2021). Additionally, Novosad and Werker (2019) traced the distribution of senior positions by nationality in the UN secretariat from 1947 to 2007 and found that China is “significantly underrepresented” (p.3). Overrepresented countries include the USA and the small and rich Nordic countries. Although there are other underrepresented member states in the UN, these countries are not comparable with China’s large territory and population size and its substantial global influence and financial contribution to the UN. China is therefore classified as an underrepresented member state in the UN.^2^

In comparison with some more developed countries, Chinese HEIs are on the periphery of training and supporting suitable talents to work in the UN. For example, Chinese HEIs only started these special programs in recent years. Nevertheless, HEIs in the USA started the degree programs and special training programs in 1898 to enhance candidates’ competencies to apply for jobs in international organizations (Yan & Zhang, 2016). Additionally, graduates from these programs in USA have high possibility to find career opportunities in international organizations including the UN. For example, over half of the 103 master students graduated from the program of international relations of George Washington University in 2013 obtained jobs in international organizations (Yan & Zhang, 2016, p.46).

US HEIs’ core position of supplying its citizens to work in the UN also benefits from the location of UN. As the UN headquarter is located in the USA, students and graduates in the USA have much more opportunities to access the UN and develop better knowledge of it. This is an advantage for US HEIs to train suitable talents and support them to work in the UN. Similar advantages are also evident in the HEIs of some other countries such as Switzerland and Austria, as both countries host regional hubs of the UN. HEIs in China do not benefit from such locational privileges because the UN regional hub in Asia and Pacific is located in Thailand.

The peripheral position of Chinese HEIs in training and supporting qualified potential candidates to work in the UN is also related with the key role of developed western countries’ in establishing recruitment standards and criteria. Although Mandarin Chinese is one of the six official languages in the UN, it is not a daily working language in the UN including its recruitment. This sets up a challenge for Chinese HEI’s endeavor to support talents to work in the UN. Furthermore, similar to the mainstream recruiting modes in the western developed countries such as USA and France, UN recruitment strongly emphasizes diversity and intercultural competencies (Liu, 2020). However, in comparison with western developed countries such as USA and France, diversity and intercultural competencies are not often emphasized in Chinese HEIs as the vast majority of graduates only seek employment in domestic labor market. Therefore, insufficient intercultural competencies and diversity awareness may become challenges for Chinese HEIs’ training and support of talents to seek career opportunities in the UN. This is possibly associated with the relatively lagged-behind internationalization of Chinese HEIs, which will be reviewed in the following section.

### Internationalization of Chinese HEIs and China’s international strategy

Despite the complexities in the definition of internationalization, there is a general consensus that the internationalization of Chinese HEIs started from an “inward-oriented” approach which refers to mainly learning from the western developed world or shifting from the former Soviet model towards a more US-oriented norm (Yang, 2013). However, with China’s rapid socioeconomic development, Chinese HEIs gradually shifted the ‘inward-oriented’ approach of internationalization to a more balanced or even an “outward-oriented” approach (Wu, 2019; Xu, 2020), through which the influence of Chinese HEIs in the world has been advanced.

It is perceived that the internationalization of Chinese HEIs is associated with the state’s international strategy. For example, Wu (2019) found that the rapid internationalization of Chinese HEIs after 2013 can be considered as part of China’s “Belt and Road Initiative”, which contributes to the global governance system including the UN. The internationalization of Chinese HEIs is also considered as an enhancement of China’s soft power, especially via the government-supported programs (Wu, 2019; Yuan, 2019). However, there is still a debate on whether at the university level, the management of HEIs has a clear awareness and understanding of such strategic roles by HEIs in enhancing the country’s soft power (Wu, 2019). This will be discussed under the questions in this paper.

## Research methods

This paper is based on two main sources of data: 53 institutional documents accessed from the official websites of Chinese higher education authorities and universities and 26 semi-structured interviews. Among the 53 institutional documents, 14 were issued by the government ministries and agencies such as Chinese Ministry of Education (MOE), China Association for Science and Technology (CAST), and China Scholarship Council (CSC). The remaining 39 institutional documents were issued by HEIs in China, including 38 issued by “double first-class” HEIs.^3^

Following the steps and principles of document analysis suggested by Gross (2018) and Bowen (2009), we conducted a preliminary search with key words including “international organizations” (国际组织), “UN internship” (联合国实习), and “China UN” (中国联合国) via Google to identify the major source of institutional documents. The preliminary search showed that the websites of MOE, CAST, and CSC were the major source of the relevant government documents. Such finding can be explained by the fact that these government departments or organizations in China have direct administrative power on HEIs or are directly related to the higher education sector. Additionally, we found that institutional documents about such special programs were predominantly issued by “double first-class” HEIs. We thus decided to use the websites of this type of HEI as a main source of documents.

After identifying the major sources of documents, we started to search the official websites of MOE, CAST and CSC for relevant documents. Fifteen governmental documents were included because they contained the relevant key words, including international organizations, UN, and UN internships. Later, one document was excluded because it replicated another document,^4^ and thus the remaining 14 governmental documents were used for data analysis.

We browsed the documents issued by HEIs from the websites of all the “double first-class” universities. At the time of document search (first half of 2020), 38 out of all the 42 “double first-class” universities’ websites had at least one institutional document related to the special programs.^5^ To ensure we did not miss any non-“double-first-class” universities, we conducted another round of web search with key terms such as “UN internships” and “UN” from China-based major search engines Baidu and Sogou so as to reduce the potential bias of Google. We found one institutional document issued by a non-“double-first-class” university.

These institutional documents cover the state and university policies to support students to pursue UN career opportunities, the activities to implement these policies, and the achievements of such activities. The number of such institutional documents (including government documents) is limited. This is possibly because the official documents of these relatively new special programs are not required to be published.

Regarding the interviews, interviewees were selected via “convenience sampling” and “snow-ball sampling” (Merriam, 1998; Martinez-Mesa et al., 2016). We used such sampling methods rather than other method such as purposive sampling because it was difficult to reach a large number of participants of such a new type of programs and we did not have access to the more macro-level data about the target population. We conducted 26 interviews among 21 students/recent graduates who had internship or work experience in the UN system with official support from Chinese higher education sector and 5 university staff members who had teaching and/or administrative responsibilities to support students/recent graduates to seek UN career opportunities (such as an international officer of a university). Among the 21 students/recent graduates, five were registered in universities outside the Chinese mainland, but all of them had received official support from Chinese higher education sector for their internships in the UN system, such as financial support from CSC. Therefore, they were also included in the analysis of this paper. For the remaining 16 students/recent graduates from the HEIs in the Chinese mainland, 14 were studying or recently graduated from “double first-class” universities. Four of the five university staff members were working at “double first-class” universities. This corresponds to the wide perception that the special programs are mainly in the elite universities in China. Due to the breakout of COVID-19 in early 2020 and the subsequent travel restrictions and lockdown policies worldwide, we were not able to meet the participants offline. We were only able to conduct interviews remotely via WeChat, the most widely used app for texting, audio, and video calls in China. With participants’ permission, the interviews were audio-recorded. We used semi-structured interviews, and the protocols were set up based on the research questions (for the interview protocols, see Appendix 1).

The interviews were conducted in Mandarin Chinese, the first language shared by the researchers and the participants. The interview recordings were transcribed verbatim by the first author, and the second author checked the accuracy of the transcription. We analyzed the institutional documents and the interview transcript in Mandarin Chinese, the original language the data were in, and also the first language shared by both authors. The other reason why we decided to do this rather than translate them into English before analysis is to ensure that the analysis can reflect the original meaning of the documents and the original voices of the participants.

Coding of institutional documents and interview scripts were conducted in three rounds (e.g., Bowen, 2009; Gross, 2018; Saldaña, 2015). Firstly, we conducted an open coding to develop a general understanding of the data contents. Secondly, a pattern coding was conducted with the main purpose to establish the major themes of the data (Saldaña, 2015; Xu et al., 2021). Thirdly, integrating both data-driven and theory-driven approaches (Fereday & Muir-Cochrane, 2006), we further identified and developed clusters of codes, logical connections, and themes. For the quotes of the interview in the manuscript, the first author translated them from Chinese to English, then the second author checked the accuracy of translation. Any disagreement was resolved by further discussion.

## Findings

From the analysis of both institutional documents and interviews, we identified four major types of special programs established by Chinese higher education sector. We also found that the policy and practice of these special programs are conforming with the center-periphery model, but these programs also have the potential to change the current peripheral status and move to the center. This section will firstly present the four major types of special programs, then illustrate how these programs are consistent with the center-periphery model, and finally explain how they might challenge the current peripheral status.

### Four major types of special programs

Based on the documents and the interviews, the special programs can be categorized into 4 types: (1) degree courses, such as Peking University’s undergraduate program about international organizations and international public policy; (2) formal collaborative projects between UN organizations and Chinese higher education, usually as set under the mutually signed Memorandum of Understandings; (3) university special funding schemes, such as special grants at University of International Business and Economics to support its students to take internships in UN organizations; and (4) university-organized UN-themed events, for example, special workshops of UN recruitment with retired senior UN officials as guest speakers (for more details of the special programs, see Appendix 2).

The first type of special programs is the formal degree courses established in Chinese HEIs at undergraduate or postgraduate level. In addition to the standard requirements such as national test scores, students are usually accepted into these degree courses based on their motivation and potential to seek employment at international organizations after graduation, plus their foreign language abilities. The degree courses are sometimes in collaboration with foreign HEIs which have good reputation for training and supporting young people to seek jobs in the UN. The curricula of these degree courses are highly related with the work of UN, such as international politics and diplomacy. The design of these courses also demonstrates a strong link with the work of UN. For example, some of the lectures are delivered by current or retired UN officials as guest lecturers, and internship at international organizations could be compulsory or optional in some courses. Foreign language training is usually essential in these courses, and students may even need to develop strong skills in foreign languages other than English. It is usually the government to finance the establishment and operation of these courses via appropriation. Most of these degree courses were established after 2015 and the first a few batches of students graduated recently. According to the interviewees, the percentage of these graduates who obtained employment at the UN was low. However, most of them have obtained job positions which require strong foreign language skills and knowledge of global affairs such as international trade.

The second type of special programs is the formal collaborative projects developed between UN organizations and Chinese higher education including the CSC. According to the interviewees, various UN organizations have developed official collaborations with Chinese HEIs and the CSC, especially after 2015. The official collaborations allow the participating Chinese HEIs and the CSC to recommend a number of students and/or recent graduates to join UN organizations as interns via relatively simpler procedures. For example, the Chinese HEIs and the CSC may conduct the initial screening while the UN organizations may make the final decisions and the allocation of internships. According to the interviewees, these formal collaborative projects are attractive to applicants, as they reduce the perceived complexity of applying for UN internships and enhance the possibility of success. However, although theoretically speaking the UN interns selected by these collaborative projects may receive stronger official support, some interviewees expressed a different feeling. They feel that once they were selected by the collaborative projects and started their internships at the UN, they no longer received much official or institutional support from the CSC and universities other than the financial support. During their internship, they received little guidance from the CSC and universities on how to work properly as interns at the UN or utilize the relevant knowledge of the UN system. Therefore, they reported that very few of them successfully obtained formal employment opportunities at the UN after their internship, which however is a core objective for the CSC and Chinese universities to establish such official collaborative projects. One interesting finding from the institutional documents and interviews is that the CSC and universities tend to prioritize collaboration with the UN organizations outside China, even though many UN organizations have branches or offices in China as well.

In the third type of the special programs, the CSC and universities officially provide financial support to students and/or recent graduates to pursue UN career opportunities. Chinese students and/or recent graduates are eligible to apply for funding from the CSC if they successfully obtained internships or other unpaid positions in the UN system. The level of financial support for interns is usually comparable to senior visiting scholars, which is attractive for applicants. Some universities also provide funding for their students to take internships at the UN, and the amount of financial support varies. The interviewees responded that the funding from the CSC or universities removes the financial burdens for taking internships at the UN. Also, as financing UN interns is a priority of the CSC, successfully obtaining grants from the CSC was not considered difficult for UN internship offer holders.

The fourth type of the special programs includes a number of university-organized events and activities aiming to introduce the UN system and support students and/or recent graduates to apply for UN careers. These activities typically included UN-themed summer schools and workshops, keynote speeches by (former) UN officials, UN-themed career events, and knowledge-sharing events between potential UN applicants and students/recent graduates who have internship experience at the UN. According to the interviewees, these activities were increasingly popular in recent years, and significantly developed students’ knowledge of the UN system including the relevant internship and job opportunities. However, these events and activities might not directly enhance students’ enthusiasm for applying for internships and job opportunities at the UN, as some interviewees explained that there are also many other non-UN themed career events which may be more attractive. This is especially the case for “double first-class” universities where most of such events and activities took place, as students from these reputed universities are more competitive in the job market and have more career choices other than working in the UN.

### Conforming to the center-periphery model

The above summary of the special programs of training and supplying talents to work in the UN by Chinese higher education sector demonstrates that these special programs are consistent with the center-periphery model. This is indicated by the late establishment of these special programs in comparison with countries in the central position, the weak follow-up support to the UN interns who benefited from these special programs, the perceived higher prestige of UN organizations outside China, and the strong emphasis on foreign languages.

The relatively late establishment of these special programs could be understood as a sign and also a reason for China’s peripheral status of training and supporting talents to apply for working in the UN. In contrast, some countries have established such programs for a longer period of time. Almost all interviewees mentioned that to their knowledge, these special programs were established in China in very recent years. The analysis of institutional documents also supports this finding as all the institutional documents obtained were later than 2015. A few interviewees also believed that these special programs somewhat were learnt from the higher education sector of the countries in the central position such as Germany, but they have limited knowledge of such special programs in other countries.

Interviews revealed that neither the CSC nor the Chinese universities provided sufficient follow-up support to the interns selected into the UN. One interviewee who benefited from the CSC and her university described the situation:

(the CSC and the university) are keen about how many people are successfully selected into (the UN) this year, but later…the follow-up is not very good…We (the interns) were not given feedback, and (the authority) did not ask whether we intend to develop a career in international organizations either… so for most (of the selected Chinese interns at UN) we simply feel ‘I just come’ (and do the internship) and go (back to China)…

Such insufficient follow-up support from Chinese HEIs and the government suggests that China is still at an early stage of supporting talents to seek career opportunities in the UN. By contrast, countries in the more central position, such as Germany, provide more follow-up support to its young citizens after they start internships to maximize their opportunities to obtain work positions at UN afterwards (Jia, 2017). An interviewee introduced that, according to a young German colleague, Germany has established a talent pool for its citizens who worked and are working in the UN (including internship). The pool can facilitate the exchange of information to seek more job opportunities in the UN. A recent study (Lu & Jia, 2021) showed that the German government has developed a more mature talent support system for German citizens to work in the international organizations including the UN. Such a system takes a “spiral approach” (Lu & Jia, 2021, p.79), through which the talents are trained and sent out to work in the international organizations, but also invited back to support further talent development and to build information exchange network. As found by Lu and Jia (2021), the German government encouraged candidates to apply for career opportunities at international organizations by holding job fairs and promoting job opportunities online. They offered tailored trainings to support potential candidates in job applications. They also launched internships, short-term trainings, and sponsored joint degree programs, especially for graduates and staff from HEIs, to develop the knowledge, skills, and competencies integral to the work in international organizations. The first stage of the spiral approach resembles the special programs set up by the Chinese government and HEIs, but the government-and-HEI collaboration on supporting talents to work in international organizations does not appear as tight as that in China. The German government further sent the talents to work in the UN with well-established liaison and coordination support and then encouraged them to return to work in Germany and establish a German network among international organizations. Lacking such strong follow-up support and a mature returning and network-building mechanism in the Chinese special programs partially explains the challenges faced by the Chinese interns in the UN. The similarities between China’s special programs and the first stage of Germany’s spiral approach suggest that China may have been learning from the center countries on supporting talents to work in the UN.

In addition, the Chinese higher education sector has a strong desire to send their students or recent graduates to take internships at UN offices in countries other than China. It is noteworthy that institutional documents show that taking internships at UN organizations or offices in China is usually not eligible to apply for financial support from the CSC. This implies that internships at UN offices in China are conceived as less prestigious than outside China, although such job opportunities can be easier to obtain by Chinese students/recent graduates. This can be further reflected by some interviewees who took internships in UN offices in China but afterwards decided to take another internship in a UN office abroad.

They explained that their internship experience in UN China offices can be used as a stepping stone when applying for internships in UN offices in other countries, which are perceived as more prestigious. The special programs’ favoring of the internships outside China implies the policy-makers’ strong intention to strengthen China’s international impact and discourse power by sending more interns to the UN systems abroad, but it devalues the UN internships in China, which can be equally important and more accessible for Chinese graduates.

Almost all interviewees and institutional documents mentioned that the special programs strongly emphasize foreign language abilities. In this context, foreign language does not only refer to English (the most widely learned foreign language in China) but also other languages especially French and Spanish. A good command of English is almost considered as a basic skill rather than an advantage for applicants of the special programs. Institutional documents also show that being able to use other foreign languages, mostly European languages like French and Spanish, would be prioritized by the selection for the special programs. It is noteworthy that apart from English, French, and Spanish, other languages including Chinese, Arabic, and Russian are also official UN languages, but they are not valued equally by the special programs. Additionally, the UN has made efforts to deal with “the pervasive hegemony of English” in its language use and language policy, and has started to embrace multilingualism for reasons including peace, security, and diversity (McEntee-Atalianis & Vessey, 2020). Despite that, the Chinese HEIs tend to attach higher value to the languages used by the center countries in both the selection criteria and curriculum design of the special programs. Such a self-initiated alignment with the languages used by the center countries chimes with the finding of a recent study that international organizations based in developed countries tend to use local languages more than those based in developing countries (Zhang et al., 2022).

Furthermore, all interviews were conducted in Mandarin Chinese, but several interviewees repeatedly expressed certain terms in English. Several were professional terms in international development and politics, official names or abbreviations of various UN organizations and departments, procedure expressions used in UN recruitment of interns, and, such as “intern,” “UNESCO,” “field offices” (meaning the non-headquarter offices of the UN), and “supervisor.” It is surprising to notice that Mandarin native-speakers express these terms in English in Mandarin-based interviews, even though all these terms have well-established translations in Chinese and can be easily expressed in Mandarin Chinese. It suggests that the dominant use of English in the UN workplace or during the UN recruitment practice has impacted the UN interns’ daily language use, similar as its strong influence in science and education (e.g., Xu, 2020). This also somewhat suggests the peripheral position of the use of Chinese in the UN and the language challenges faced by the Chinese higher education sector in training and supporting qualified candidates to work in the UN.

### Striving to move to the center from the periphery

This study also identifies that the Chinese higher education sector is striving to move from the peripheral to a more central position via the special programs. In other words, these programs do not challenge the center-periphery division itself, but they strive to change China’s position within the center-periphery model. This can be reflected by the special programs’ consistency with China’s globalization strategy of moving from the periphery to the center of international society (Cheng & Zhai, 2021) and also the integration of these special programs into the internationalization of Chinese higher education.

All the government and university documents explicitly state that these special programs are established as a response to the country’s internationalization strategy, which includes an important objective to expand China’s global influence by increasing its presence in multilateral international organizations such as the UN (Cloke, 2020). For example, the Ministry of Education of China (2017, p.1) stated at the beginning of the document “…accelerating the training and supply of university graduates to seek internships and jobs at international organizations plays an important role in enhancing the country’s influence on making international rules.” Similarly, all interviewees responded that universities and the CSC established these special programs fully or partially because of these programs are in line with the country’s internationalization strategy. For example, an interviewee recently completed internship at UN commented:

These special programs are part of China’s internationalization strategy. I feel they aim to promote China’s discourse power in international affairs …including in international organizations… (but) they are only part of China’s internationalization strategy because there are other (objectives and actions) such as international aid…

This information suggests that China is taking efforts to move towards the center, and the special programs are part of these efforts. There is also evidence that these special programs can support China’s global strategy of moving from periphery to the center and increase its global influence in a less sensitive approach. This corresponds to the existing studies that soft power is sometimes less sensitive and more effective in increasing a country’s international influence (e.g., Yuan, 2014). For example, an interviewee who is currently taking an UN internship commented:

Via UN, such a developmental system (global development platform), providing international aid to developing countries such as the third world countries…is more multilateral, and via such multilateral platform…the areas of collaborations could be wider, and possibly less politically sensitive. This would be useful to improve the impact of the foreign aid, and it is also good for implementing China’s foreign aid policies.

“Global reputation” and “going-out” were mentioned by several interviewees. These two terms are highlighted in the process of Chinese higher education internationalization (e.g., Xu, 2020). According to the majority of interviewees, Chinese universities established these special programs not only as a response to China’s national strategies of internationalization, but also as a means to accelerate the internationalization of Chinese higher education and promote the global reputation of universities. As commented by interviewees, having graduates seeking career opportunities in the UN can bring advantages to universities. For example, it may attract more competitive applicants at the admission stage and establish a more internationally recognized alumni network. An interviewee who was a senior university management staff further introduced that the establishing these special programs is often considered as an indicator of the internationalization of HEIs, and graduates’ successful application of UN jobs can bring pride to the universities, because UN is widely considered as a well-reputed employer. Some other respondents argued that sending students to take internships at the UN would enable people in other countries to have a better knowledge of Chinese universities and make the universities better recognized and accepted internationally, because of UN’s global reputation. An interviewee used an analogy of business world:

Similar to a company, whether it is Top 500 locally, nationally, or globally is entirely different. Only regional influence is not enough. International influence is also needed. For a university (having more students and graduates in the UN) it is multidimensional development. More people will know the university, and the university will become more popular, and get more development (opportunities) and government support. This is especially for (universities in) second tier cities…which are not globally well known.

The analysis of these interviews and institutional documents suggests that establishing these special programs demonstrates the country and the universities’ ambition of and dedication to moving towards the center, as these special programs are expected to enhance China’s influence in international affairs, and the universities’ global reputation. In other words, the findings provide evidence that these special programs are consistent with China’s overall strategy of internationalization, which is to move from the periphery to the center. Meanwhile, it reflects Chinese HEIs’ desires to move to a more central position in the internationalization of higher education. Therefore, these special programs demonstrate that a close state-university relationship may still exist in China, and the HEIs are aware they can act as a soft power to enhance the country’s global influence.

## Conclusion

In the center-periphery context, the special programs are connected with continuing global inequalities, as reflected by some conforming practices in the special programs. Also, alternative dynamics exist in the special programs—China and Chinese higher education sector have been striving to move from the periphery to the center, although such a move does not challenge the existing center-periphery division itself.

When establishing the special programs, Chinese higher education acknowledged its peripheral position in the world. As shown in the previous sections, these special programs are very new and still at an early stage if compared with the countries in the central position. The special programs strongly emphasize the conforming practice to the current recruitment regulations and practices of the UN rather than making changes to them, even though China is one of the five permanent member states of the UN Security Council. The dominance of foreign languages (especially English) in the UN recruitment practice can be observed in the analysis, which is also a dimension of the center-periphery inequality (Xu, 2020). Such dominance still exists even Chinese is also an official language of the UN since its establishment in 1945.

Nevertheless, alternative dynamics demonstrate China and its higher education’s desires and practices are not accepting the current peripheral status. These special programs are established to achieve China’s global strategy which emphasizes moving to the center of international platform via multilateralism and stronger global influence. They are also consistent with the internationalization of Chinese higher education, which is part of the country’s global strategy.

The special programs could be conceptualized as a top-down process. It starts from the central government’s call for enhancing China’s influence in and via international organizations. It then evolves into the responsive actions of the higher education authorities and universities to establish these special programs and to proactively support talents to seek jobs in the UN. These notions and practices are different from just accepting the peripheral position and passively staying inferior to the global center (Xu, 2020). Instead, it demonstrates the country’s desire to move towards the global center. The special programs in Chinese higher education sector and China’s global strategy are working in tandem to achieve this goal.

As a pioneering study in this field, this article enriches the literature of higher education internationalization by using the center-periphery model to interpret the special programs for supporting Chinese talents to seek career opportunities in the UN, which is an underexplored research topic. It also promotes dialogues on the relationship between national internationalization strategy and the globalization of higher education, especially in China. Another unique contribution of this study lies in the rich, first-hand qualitative data from interviews with recent beneficiaries of the special programs. The insights provided by those young “insiders” of the UN establish a valuable knowledge foundation for future studies.

This study is of course not free of shortcomings. As these special programs have just been set up for a few years and it takes time for the supported/sponsored university graduates to find their pathway and build up their career in the UN system, it can be too early to comprehensively evaluate the achievements, effectiveness, and limitations of these special programs. Future research efforts can track the talents’ career development and Chinese staff representation in the UN as the special programs develop in a longer term and explore reasons for the underrepresentation of Chinese (and also some other countries) nationals in the UN. We are also unable to conduct an in-depth comparative analysis of similar special programs in other countries due to the difficulty in collecting the relevant data. Therefore, we call for future studies to compare UN talent-support systems across countries. Such empirical studies will deepen our understanding of the intricate relations between higher education internationalization, state strategies of internationalization, and the world order manifested by international organizations.

## Appendix 1 The representative special programs supporting talents to seek UN career opportunities

In the main body of the article, four types of special programs in Chinese higher education supporting talents to seek UN career opportunities are mentioned. Due to the space limitation, we are unable to introduce all of them in detail. This appendix introduces a few representative programs of the four types in more detail so that readers may have a better understanding of these special programs.

1. Bachelor's degree program in international organizations and international public policy (IO&IPP) at Peking University (PKU)

As a formal degree program with an objective to support talents to seek UN career opportunities, PKU’s IO&IPP started to recruit undergraduate students in autumn 2018. Therefore, the first batch of job market candidates will complete their studies in the summer 2022. As shown in the recruitment prospectus and program introduction, IO&IPP is a degree program under PKU’s School of International Relations.

It is the earliest degree program in China which focuses on the roles of international organizations in the formation, evaluation, and dissemination of global public policies and has strong objective to improve the competencies of its graduates to work in international organizations. It is clearly described that a major employment prospect for graduates from IO&IPP is intergovernmental organizations including the UN.

In addition to the clear employment prospect, IO&IPP has two features in supporting talents to seek UN career opportunities: (1) a substantial proportion of its teaching staff have work experience in international organizations including the UN; and (2) the program provides its students with internship opportunities at major organizations, departments, and duty stations of the UN. It is unable for this article to seek further data of how many students have been offered with such internship opportunities because the earliest interns from IO&IPP would be in late 2021 or 2022, after the submission of this article (only final year undergraduates are eligible for UN internships).

Source of information: PKU IO&IPP recruitment prospectus and program introduction, available at https://www.sis.pku.edu.cn/undergraduate/notice_18020/1307142.htm, accessed on 30 March 2022, in Chinese.

Similar degree programs include but are not limited to the following:

1. School of International Organizations, Beijing Foreign Studies University. https://sio.ibfsu.cn/#
2. School of International Governance Innovation, Guangdong Foreign Studies University. https://sigi.gdufs.edu.cn/gywy/zsxx.htm
3. Research Center for the United Nations and International Organizations, Fudan University. https://iis.fudan.edu.cn/6782/list.htm

2. Formal collaboration between the UN and Chinese higher education: UNESCO-China Scholarship Council (CSC) internships

According to a Memorandum of Understanding between UNESCO and CSC, UNESCO will accept a designated number of Chinese interns recommended and supported by the CSC every 2 years. This collaboration started in 2014, and the most recent selection was in 2021 (internships expected to start in 2022 following selection procedures). To the authors’ best knowledge, this is the earliest formal collaboration between the UN and CSC.

According to the program introduction cross-published by the Chinese Ministry of Human Resources and Social Security (MOHRSS), 21 UNESCO internships were planned to be offered to eligible Chinese nationals via this UNESCO-CSC collaboration for the most recent selection (2021–2022). Eligible candidates must be current postgraduate students or postgraduate degree holders within one year of graduation. Candidates need to submit their applications via universities or designated Chinese embassies (if they are temporarily studying overseas at the time of application), and CSC will not accept applications directly from individuals. Eligible candidates must be proficient in English and Chinese, and knowledge of other foreign languages are highly desirable.

CSC will organize written tests and interviews to select candidates and produce a list of recommended candidates to UNESCO. UNESCO will confirm the candidates to be selected. The successful applicants are expected to take the internships for 6–12 months, and the CSC will cover the cost of the whole duration of their internships at the level equivalent to senior visiting fellow (EUR 1,200 at UNESCO’s headquarter in Paris), plus the return flight tickets to and from the internship location. However, it is noted that although the internship locations vary, the CSC does not support interns at UNESCO’s office in China.

Source of information: MOHRSS (2021) Notification of 2021 the UNESCO Internship Project. Available at: http://io.mohrss.gov.cn/a/2021/09/23/10935.html, accessed on 29 March 2022, in Chinese.

In addition to the UNESCO-CSC collaboration, CSC has established similar formal collaborations with some other UN organizations. For example, in 2021, CSC also sponsored 8 Chinese nationals to pursue internships at UNIDO. See MOHRSS (2021) http://io.mohrss.gov.cn/a/2021/09/23/10936.html accessed on 5 May 2022, in Chinese.

3. Special grants supporting students from University of International Business and Economics (UIBE) to take internships in UN organizations

UIBE and United Nations Industrial Development Organization (UNIDO) have established institutional cooperation. Such cooperation will allow UIBE to recommend 2 interns each semester to take internships at UNIDO for around 3–6 months with financial support from UIBE’s. This is a typical university special scheme to support talents to seek UN career opportunities.

According to the 2017 advertisement of UIBE-UNIDO internship (UIBE, 2017), the successful applicants were financially supported by the special grants such as the university funded Special Bursary for Training and Recommending Global Governance Talents (*Quanqiu Zhili Rencai Peiyang Tuisong Gongzuo Zhuanxiang Jijin*). If the successful applicant is an undergraduate student, he/she is also eligible to be supported by the university funded Special Bursary for Undergraduate Overseas Study (*Benkesheng Haiwai Liuxue Zhuanxiang Zijin*).

To be eligible for the support, the applicants must be Chinese national registered with UIBE as a senior undergraduate student or postgraduate student, and be proficient in English (fluency in second foreign language is desirable). The grants aim to support students taking internships at UNIDO although in practice, internships at other UN organizations may also be supported. The exact amount of financial support is not available to the public, while the applicants may know the approximate amount via university internal sources. The university will also support the visa application if necessary so that interns do not need to undertake the relevant admin costs. Interviewees who have benefited from these special grants mentioned that the amount of financial support they obtained is sufficient for their reasonable expenses during the whole internship period.

Source of information: UIBE (2017). UNIDO internship project 2017. Available at: https://aeo.uibe.edu.cn/front/showContent.jspa?channelId=1709&contentId=5052, accessed on 30 March 2022, in Chinese.

A similar special grant to support university graduates to pursue internships is the joint grants provided by the University of Shanghai for Science and Technology (USST) and Shanghai Municipality. The eligible applicant for the special grant must be a student of USST and have offer of UN internship lasting 3–12 months. The grants are jointly provided by USST and Shanghai Municipality, although there is no detailed information of their exact shares. See USST (2021) at: https://ie.usst.edu.cn/2022/0222/c8762a265574/page.htm, accessed on 5 May 2022, in Chinese.

4. International Organizations Talents Training Camp (IOTTC) at Tsinghua University (THU)

Organized by the Career Centre of and other internal units of THU, the International Organizations Talents Training Camp (*Guoji Zuzhi Rencai Xunlianying*) started in 2017, and the second camp took place in 2019. This is a typical university-organized UN-theme event on campus.

The introduction of the 2nd IOTTC at THU clearly states that supporting talents to seek UN career opportunities is a university internationalization strategy and also corresponds to the country’s strategy of internationalization. The 2nd IOTTC lasted for a few days in late 2019 with intensive training on UN recruitment, UN career paths, China’s contribution to UN and global governance, and resources and opportunities for seeking UN careers. In addition to university teaching staff, the trainings are delivered by a wide range of external professionals such as senior UN officials and diplomats.

To be eligible to apply for the IOTTC, the candidate must be a current student of THU and have strong foreign language skills. Another essential criterion is that the candidate must have strong intention to work at international organizations including the UN after graduation from THU; however, there is no public information on how to assess the career intention of candidates. Successful applicants do not need to pay for any cost, even though THU may need to afford the relevant expenses such as inviting external professionals to deliver trainings.

Source of information: THU Student Association for Global Governance and International Organizations (2019) Do not miss the 2^nd^ IOTTC. Available at: https://goglobal.tsinghua.edu.cn/news/announcement.cn/b6WvKvDZkK, accessed on 29 March 2022, in Chinese.

A few similar UN-themed career events organized by Chinese HEIs are listed below:

1. International Organization Experience Week, Xiamen University. https://jyzd.xmu.edu.cn/2021/1215/c18717a445541/page.htm
2. UN Organization Career Presentation and Consultation, Wuhan University. https://news.whu.edu.cn/info/1007/67065.htm
3. International Organization Career and Internship Training Camp, Nankai University. https://fcollege.nankai.edu.cn/2020/1110/c7309a329610/pagem.htm

To conclude, there are many similar programs in addition to the selected programs as mentioned above. These programs are selected for introduction largely because of their relatively early establishment, supported by high-level authorities, and/or based in widely known universities. It is noted that these special programs are somewhat affected by COVID-19 so that we have relatively less information about them in 2020. In the future, we may consider conducting in-depth case studies of a few selected special programs.

## Appendix 2 List of main interview questions

We have produced a list of major questions for the interviews. As the interviews were semi-structured, not all of these questions were asked. In addition, interviewees have full rights and freedom to refuse answering any question during the interview; therefore, not all of them answered all the questions asked by the interviewers (the authors). Also, to ensure the questions not to impede the conversations, they may not be asked in the exact contents, sequences, and formats as shown below. In short, this list only aims to provide brief information of the interviews so that readers may have a better understanding of the qualitative information obtained from the interviews.

1. Through what channels did you obtain the information of UN career opportunities?
2. Through what channels did you apply and obtain the UN career opportunities?
3. To your knowledge, what are the main efforts that the government and universities have made to support young talents to seek UN career opportunities?
4. From your knowledge and perhaps also your personal experience, what are the challenges and difficulties of these special programs?
5. In your opinion, what are the main advantages enabling you to get such an opportunity in the UN?
6. What (how) do you think the special programs benefit you to get such an opportunity in the UN?
7. Why do you use these special programs to seek UN career opportunities?
8. In your opinion, why universities support talents to seek UN career opportunities?
9. In your opinion, what are the relationships between these special programs and higher education internationalization? Or perhaps there is no relationship?
10. In your opinion, what are the relationships between these special programs and the country’s internationalization? Or perhaps there is no relationship?

If the interviewee is a university staff member instead of a student/recent graduate, above questions 2, 5, and 6 will be readjusted accordingly. For example, question 5 will be readjusted into “what are the strengths enabling your students (students you know) to get UN career opportunities?”.

In the actual interviews, the term “UN” is sometimes interchanged with “international organization” as the UN is the most influential international organization and all the student/graduate interviewees were successful applicants for UN career opportunities. Although the questions require some in-depth thinking to be properly answered, this does not become a challenge for the interviewees because they are well-educated and have sufficient ability to understand the questions and express their answers clearly.

In addition, since the interviewees have UN career experience and are beneficiaries of the special programs, these questions are not new to them. Instead several interviewees commented that they are also interested in these questions and have already thought about them in the past on their own. Some interviewees even commented that the interviews would be a good opportunity for them to share their thoughts with the research experts (the authors). That is an important factor to ensure the quality and trustworthiness of the interviews.

## Footnotes

1 In theory, taking internships at the UN will not automatically lead to employment opportunities at the UN after the internship. However, UN interns have chance to know the real day-to-day working environment of the UN and may enhance their competencies to work in the UN during their internships. Therefore, in practice, UN interns usually have more advantages when applying for employment opportunities after their internships. That is why UN internships are also considered as career opportunities.

2 The variations in the number of employees at UN are mainly due to measurement disparities and high mobility of UN employment. In terms of measurement, the UN System Chief Executives Board for Coordination (2021) reports the number of Chinese employees in the wider UN, while Liu and Xiao (2018) report the number of Chinese employees in the UN secretariat (headquarter) only. However, regardless of the measurement disparity, China is an under-represented member state in terms of the number of employees at the UN, which is an agreed conclusion from all sources. Also due to the rapid changes of UN’s work, the UN recruits a lot of temporary employees, which may lead to sharp fluctuations of its workforce from time to time. For more details of UN employment and recruitment, see Liu (2020).

3 The “double first-class” strategy, initiated in 2015, is a Chinese higher educational policy which aims to develop a number of first-class universities and first-class subjects. In some way, it can be considered as an update of the widely-known “211” and “985” projects (Xu, 2020).

4 The reason for the complete replication is that the same documents are posted on the websites of different government organizations.

5 Such browsing of university websites was plausible at the time of search because the number of “double first class” universities in China was not large (only 42).
